# Supplementary material for: Melatonin Exerts Prominent, Differential Epidermal and Dermal Anti-Aging Properties in Aged Human Eyelid Skin Ex Vivo
Source: Int J Mol Sci. 2023 Nov 4;24(21):15963. doi: 10.3390/ijms242115963 (PMC10647640; doi:10.3390/ijms242115963)
Supplement: Supplementary file 1 [file ijms-24-15963-s001.zip › ijms-2637524-supplementary.pdf]

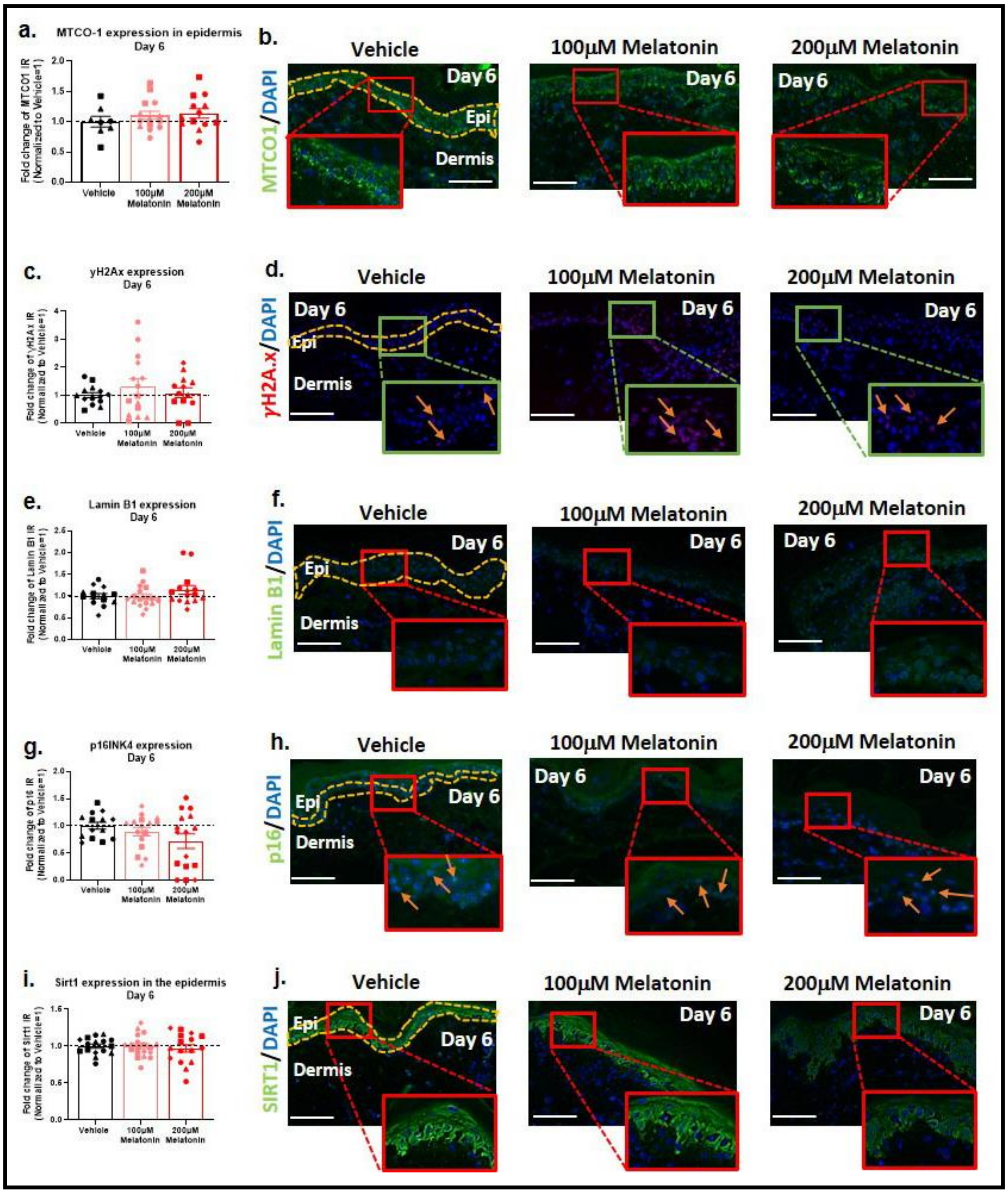

Supplementary Figure S1. The anti-aging effects of melatonin are not indiscriminate. (a-b) Treat-ment of 200 µM melatonin showed upward trend of MTCO-1 in epidermis on day 6. Quantitative (immuno-)histomorphometry and representative pictures of MTCO-1 expression. Mean +/- SEM; n=12-17 not consec-utive skin sections from 4 independent donors; Mann-Whitney test, not significant. **(c-d)** γH2Ax expression after 6 days of culture in the lower two cell layers of the epidermis after treatment with 100 µM and 200 µM melatonin. Quantitative (immuno-)histomorphometry and representative images of γH2Ax expression. Mean ± SEM; n=13-20 non-consecutive skin sections from 4 independent donors; Mann-Whitney test. **(e-f)** Lamin B1 expression after 6 days of culture across the entire epidermis after treatment with 100 µM and 200 µM melatonin. Quantitative (immuno-)histomorphometry and representative images of lamin B1 expression. Mean ± SEM; n=14-18 non-consecutive skin sections from 4 independent donors; Mann-Whitney test. **(g-h)** p16INK4 expression analyzed in the two lower layers of the epidermis after treatment with 100 µM and 200 µM melatonin on day 6. Quantitative (immuno-)histomorphometry and representative images of p16INK4 expression. Mean ± SEM; n=13-20 non-consecutive skin sections from 4 independent donors; Mann-Whitney test. **(i-j)** SIRT-1 expression in the entire epidermis after 6 days of culture treated with 100 µM and 200 µM melatonin. Quantitative (immuno-)histomorphometry and representative images of SIRT-1 expression. Mean ± SEM; n=16-18 non-consecutive skin sections from 4 independent donors; Mann-Whitney test. Epi: epider-mis. Scale bar: 100um
